# Vaccination in Germany

**Published:** 1898-01-22

**Authors:** 


### VACCINATION IN GERMANY.

The *British Medical Journal* gives a *résumé* of the returns in regard to small-pox and vaccination which have been recently issued by the Statistical Department in Germany for the year 1895. They exhibit the remarkable spectacle of a vast population, the land frontiers of whose country are conterminous with those of people among whom vaccination is neglected and small-pox prevalent, enjoying an unexampled exemption from that disease. The returns show that the immunity of the German people is attributable not so much to the efficiency of the primary vaccination as to the practice of compulsory revaccination of all children in their fourteenth year—that is, at the end of the period of obligatory school attendance. The total number of deaths in the Empire from small-pox was but 27, distributed among 18 localities. Of the 27 cases 14 occurred within a few miles of the frontier. Eleven of the deaths were in unvaccinated infants under two years of age, and three others were in children under ten who were certainly unvaccinated. Vaccination in Germany is evidently very carefully carried out, the percentage of successes in primary vaccination ranging from 99·68 in the Province of Schwabe to 92·03 in Lübeck.

The success of primary vaccination, however, cannot be accepted unreservedly as a criterion of the protection of a district. Perhaps a better test is to take the inverse proportion of success in revaccination. The percentage of successes in revaccination ranged from 99·63 in the Black Forest to 57·32 in Hamburg, showing how effectually the primary vaccination of the population had been carried out in that city. Eleven deaths, or about one to 100,000 infants, were alleged to have been caused by vaccination. In seven of these the first inflammatory symptoms appeared three or four weeks after the operation, and were evidently due to subsequent infection from insanitary surroundings, and in one that followed closer after vaccination the arm had been bandaged by ignorant parents with a dirty rag. So far so good; but we think that it is rather stretching a point to insist on the distinction which must be drawn between vaccinal-erysipelas and the erysipelas-after-vaccination which occurs in the second or third week, and to assert that for this latter the vaccinator is in no way responsible. No doubt this is literally correct, and "the vaccinator" is in no way responsible, but the vaccination is responsible, and that is really the matter of interest. If the vaccinator is at fault that is a thing which can be corrected, but if the mischief is due to the surroundings of the patient we must, we fear, expect a certain number of cases of erysipelas to follow the operation however perfectly it may be performed—unless, indeed, the experiments now being made in regard to protective dressings should show a way of escape.

